# HIV KNOWLEDGE AND INFORMATION ACCESS AMONG FEMALE CANCER SURVIVORS IN NIGERIA

**DOI:** 10.1093/geroni/igad104.0406

**Published:** 2023-12-21

**Authors:** Candidus Nwakasi, Darlingtina Esiaka, Chizobam Nweke

**Affiliations:** University of Connecticut, Storrs, Connecticut, United States; University of Kentucky, Lexington, Kentucky, United States; St George’s University, St. George, Saint George, Grenada

## Abstract

While Nigeria remains heavily burdened by HIV, especially among Nigerian women, there is also a rapidly increasing incidence and prevalence of cancer among aging women in Nigeria. growing. A body of research posits that HIV can be detrimental to cancer survivorship experiences including a worsened health outcome for cancer survivors. Further, HIV-relevant healthy behaviors such as sexual and reproductive health may be limited by a knowledge gap in the relationship between HIV and cancer, thus, increasing the risk of negative health outcomes among cancer survivors. This stresses the importance of HIV prevention and health promotion among cancer survivors in Nigeria, and one of such prevention strategy is the use of HIV health information. This study employed a qualitative descriptive method to assess HIV knowledge and information access among female cancer survivors in Nigeria. Thirty female cancer survivors in Nigeria were recruited and interviewed using a semi-structured interviewing format. Findings based on their responses indicated their knowledge about the connection between HIV and cancer. The themes are comparing HIV and cancer, views on the effect of HIV on cancer, and seeking HIV health information. Our findings highlight the need to utilize tailored public health education interventions for the growing number of cancer survivors in Nigeria to reduce the risk of poor health outcomes in this population.

